# Integrative function of proprioceptive system in the acute effects of whole body vibration on the movement performance in young adults

**DOI:** 10.3389/fspor.2024.1357199

**Published:** 2024-04-09

**Authors:** Olga Maslova, Natalia Shusharina, Arseniy Videnin, Vasiliy Pyatin

**Affiliations:** ^1^ Neurosociology Laboratory, Neurosciences Research Institute, Samara State Medical University, Samara, Russia; ^2^ Baltic Center for Neurotechnologies and Artificial Intelligence, Immanuel Kant Baltic Federal University, Kaliningrad, Russia; ^3^ Physiology Department, Samara State Medical University, Samara, Russia; ^4^ Neurointerfaces and Neurotechnologies Laboratory, Neurosciences Research Institute, Samara State Medical University, Samara, Russia

**Keywords:** integrative proprioceptive system, acute WBV, acute FMSE, motor patterns, center of pressure, young adults, postural control

## Abstract

**Background:**

The proprioceptive system coordinates locomotion, but its role in short-term integration and recovery of motor activity in imbalance of motor patterns and body remains debated. The aim of this study is investigating the functional role of proprioceptive system in motor patterns and body balance in healthy young adults.

**Methods:**

70 participants (aged 20.1 ± 0.3) were divided into experimental groups EG1 (*n* = 30), EG2 (*n* = 30), control group (CG, *n* = 10). EG1 performed single WBV session on Power Plate (7 exercises adapted to Functional Movement Screen (FMS). EG2 performed single session of FMS Exercises (FMSE). CG didn't perform any physical activity. All participants performed pre- and post-session of FMS and stabilometric measurements.

**Results:**

FMS total score in EG1 increased by 2.0 ± 0.2 (*p*_0_*_ _*< 0.001), this was significantly differed (*p*_0_*_ _*< 0.001) from EG2 and CG. Acute effects of WBV and FMSE on rate of change and standard deviation (SD) of pressure center (COP) were shown in all groups during Static Test (*p*_0_*_ _*< 0.01). SD increased (*p*_0_*_ _*< 0.01) in Given Setting Test in EG1 and EG2, and in Romberg Test (*p*_0_*_ _*< 0.001) in EG1. Length, width and area (*p*_0_*_ _*< 0.01) of confidence ellipse, containing 95% of the statokinesiogram points, decreased in Static Test in EG1; width and area (*p*_0_*_ _*< 0.01) decreased in EG2 group. Significant (*p*_0_*_ _*< 0.01) decrease in Given Setting Test was in EG1, and significant (*p*_0_*_ _*< 0.01) increase was in Romberg Test (open eyes) in CG. Maximum amplitude of COP oscillations: significantly (*p*_0_*_ _*< 0.01) decreasing along *X* and *Y* axes in EG1 and EG2, and along *Y* axis in CG during Static Test; along *Y* axis (*p*_0_*_ _*< 0.01) in all groups during Given Setting Test. Significant differences were identified (*p*_0_*_ _*< 0.01) in calculated energy consumption for COP moving during all stabilometric tests. However, inter-group differences in COP after acute WBV and FMSE sessions have not been identified.

**Conclusions:**

Acute WBV session eliminates the deficits in motor patterns which is not the case after acute FMSE session, which, according to our integrative movement tuning hypothesis, is due to high activation of integrative function of proprioceptive system. Efficacy of WBV and FMSE on COP performance indicates a high sensitivity of postural control to different levels of proprioceptive system activity.

## 1 Introduction

The main target of *Whole Body Vibration* (WBV) is the nervous system and musculoskeletal system (1–12). The WBV physical factors such as mechanical vibration frequency, acceleration, and gravitational force initiate in an authentic manner proprioceptive activation that elicits a similar in frequency reflex contractions of skeletal muscles and, driven by the integrative function of the proprioceptive sensory system, recruitment of vegetative reactions, neuroendocrine responses, and positive immune response and osteogenic transformation (7, 13). In addition to WBV, targeted vibration interventions are used on the body areas for the purpose of myofascial relaxation or WBV massage (14).

The WBV activation of the proprioceptive system begins at the level of muscle spindles and integrates in sensorimotor cortex (15), and has been previously shown integrates at different levels of the central nervous system (CNS), regulating executive brain functions according to levels of proprioceptive stimulation. The integrative properties of the proprioceptive system at the CNS segmental level are manifested by the tonic vibration reflex (16, 17) and tendon reflexes (6, 18) with recruitment of the maximum number of motor units of skeletal muscles (16, 19). At the stem level, proprioceptive interneurons integrate vegetative functions (blood circulation, respiration) (20–22), nociception (9) and balance (23) during WBV. The integrative properties of the proprioceptive system at the level of the hypothalamus elicits neuroendocrine responses, and at the level of sensorimotor cortical areas (M1) (15, 24) participates in the control of motor activity, improves cognitive function and psychological well-being such as mood (25, 26).

Due to the integrative properties of the proprioceptive system, the WBV-initiated physiological responses of the CNS and musculoskeletal system have numerous examples of WBV applications in sport (8, 27–29). There are the publications proving rapid increases of muscle strength in upper and lower limbs (3) as well as flexibility (30) and body balance by the WBV impact including in elderly adults (2, 4). The authors (1) previously emphasized that most of the studies conducted to date have focused on the acute and chronic effects of WBV on neuromuscular activity.

Studies have shown the effectiveness of WBV in both acute and long-term vibration therapy protocols. In particular, the short-term and long-term WBV sessions have a proven rehabilitative effect in many fields of medicine, and this is a well-proven fact (31–33). Recently, the acute positive effects of a single WBV session on the physical fitness, flexibility, body balance and cognitive performance in healthy individuals, as well as on target skeletal muscle endurance (34), in sport applications (29) have received much attention in the literature. Earlier in our work, we found that a single WBV exercise on the Power Plate vibration platform significantly increases expiratory airflow velocity in healthy subjects (35). When WBV is complexed with other training methods, greater training or rehabilitation effects are achieved (14, 36, 37). However, in the most studies or reviews, the outcome of improving muscle function with WBV is maximal muscle strength and power (38–40). The objectification of WBV effects is correlated with informative indicators such as maximal voluntary isometric contraction strength, low and high isokinetic concentric and eccentric contraction strength, and acceleration time (5).

Two things can be emphasized because of the literature review. Firstly, WBV, regardless of the goals of physical improvement or rehabilitation, utilizes standardized form of WBV exercises. The main conditions of WBV are frequency, amplitude of vibration exposure, duration and number of exercises per session and number of sessions (1). Second, the efficacy of WBV is not quantified by parameters of complex motor patterns (41, 42). Thus, the result of a WBV sports training is an increase in athletic performance, such as jump height, after both WBV acute and chronic exposure (8). In rehabilitation, motor recovery after musculoskeletal injuries (43) or after central motoneuron function damage because of stroke (44) is assessed by subjective scales of the Action Research Arm Test (ARAT) (45) and the Fugl-Meyer scale (46). At the same time, the motor patterns are complex integrative processes regulated at different CNS levels. Until now, they have not been the object of research in the WBV application technologies. However, Functional Movement Screen (FMS) in identifying the motor pattern impairments and the restorative Functional Movement Screen Exercises (FMSE) program are known to be an effective approach to sports injury prevention (47, 48).

FMS offers a set of validated tests of functional movement patterns diagnosis (49), which we believe may represent a promising technique to be integrated with the WBV technology. FMS is a validated musculoskeletal assessment system for identifying movement deficits in novice athletes to predict the risk of injury. However, it is important to emphasize that features of professional sport also cause musculoskeletal impairments. A typical source of motor imbalance may be loading asymmetry, for instance in elite short-track athletes (50). Therefore, the technology to quickly and effectively elimination the disorders in motor patterns and body balance in a wide range of population involved in sporting activity is relevant. For this purpose, FMS was designed by Cook G. to assess movement functionality, consisting of seven fundamental movement patterns (tests) that require a balance of mobility and stability (including neuromuscular/motor control) (47, 48). FMS is often used to assess the risk of injury, taking a score of ≤14 as an indicator of a person's poor fitness and body shape. A score of ≤14 posed more than twice the risk for musculoskeletal injuries (51). A few studies have shown a positive effect of the restorative FMSE program after 6 and 8 weeks of the corrective training in both young (52) and adult athletes (53, 54).

Typically, movement deficits are combined with impaired statistical body balance and deviations in holding upright posture, good posture regulation is thus the basis for almost every movement (41). Body balance control is a complex integrative function of different brain networks, which among other things automatically condition the connection between postural and movement control (42). An integrative biomarker of postural control is the trajectory of the center of pressure (COP)—known as the stabilogram—while a person is standing quietly. This quantification has many important applications, such as early detection of balance deterioration to prevent falls (55–57). Analyzing variations in CОP is the one way to quantify the coordinates COP in time that trace a trajectory along time on the XY plane (57) and postural stability (56). However, it is believed that new postural and balance biomarkers are required for diagnostic purposes and rehabilitation decision making (57).

Positive aspects of FMS and effects of the FMSE program to correct movement deficits are usually achieved within a few weeks (49). The impact of a single FMSE session on the elimination of the motor deficits diagnosed by the FMS method has not been shown in the literature; the focus continues to be on the traditional approach in the FMS and FMSE areas (58–60).

To date, there are no literature data on the leveraging of the short-term WBV sessions for the correction of deficits in the fundamental movements and body balance diagnosed by FMS and stabilogram. It has been no comparative study of the acute effects of WBV vs. FMSE on the correction of the motor deficits in young adults there. The diagnostic capabilities of FMS and stabilogram to assess the physical condition of young people are known in the literature, as well as the capabilities of the long-term corrective FMSE (12, 61, 62). However, it was shown that in case if the FMSE training is not continued continuously in the high school baseball players, then the FMS scores decrease (61).

Until recently, motor patterns and postural control of conventionally healthy young adults have not been investigated under conditions of targeted activation of the proprioceptive brain system using WBV technology. Given the available information about the key role of the proprioceptive sensory system of the brain and its integrative role of other integrative CNS systems in different WBV strategies, we suggest that the study may be based on the integrative movement tuning hypothesis. We hypothesize based on this analysis that the different levels of activation of the proprioceptive system of brain may be of key importance in the regulation and restoration of motor patterns and body balance. The aim of this study is to determine whether the peculiarities of different levels of integrative influence of the proprioceptive system on control systems of motor activity, in particular motor patterns and postural control in conditionally healthy young adults.

## 2 Materials and methods

### 2.1 Study design and participants

The study involved seventy students (21 males and 49 females) aged 18–24 years old (*M* = 20.1; SD = 0.3) at Samara State Medical University (Table 1). All participants signed the informed voluntary consent form to participate in the experiments. The study was conducted according to the Declaration of Helsinki ethical standards and approved by the Ethical Committee of Samara State Medical University (protocol N48/17.11.2021). Study participants were randomly divided into two experimental groups EG1 (*n* = 30) and EG2 (*n* = 30) and one control group CG (*n* = 10). Randomization of the study participants was carried out by the envelope method. They were offered to choose one of 70 envelopes, each containing the number of one of the three groups. Prior to the study, all participants read and signed the informed consent form. In addition, participants in EG1 were informed and confirmed in writing form that they had no contraindications for performing exercises on the Power Plate platform. One week before the experiment, the subjects received an information letter by email in which they were instructed to avoid the intake of stimulants such as coffee and alcohol for at least 12 h before the session, and to wear comfortable clothing during the experimental sessions.

**Table 1:** Demographic characteristics of the participants.

Number of participants		70
Gender	Male	21 (30.0%)
	Female	49 (70.0%)
Age, years		20.1 ± 0.3
Body weight, kg		64.1 ± 1.2

Inclusion and exclusion criteria were determined. The inclusion criteria were individuals (a) of both gender, (b) aged 18–24 years old, (c) with normal vision, (d) with normal hearing, (e) not taking the medications that influence the balance system, (f) with absence of vestibular or neurologic disorders, (g) with absence of lower extremity injuries, spinal disorders, (h) who signed the ICF, (i) who had not the previous experience with WBV prior to participating in the experiment.

The exclusion criteria were individuals with (a) a history of vertigo or dizziness, (b) presence of vestibular or neurologic disorders, (c) uncorrected visual problems, (d) sustained lower extremity injuries, spinal disorders, (e) taking the medications that influence the balance system, (f) hearing loss, (g) acute/chronic ear infections, (h) use of a pace-maker, (i) surgeries with implantation of metallic material, (j) peripheral vascular disease, (k) recent post- operative period, (l) diabetes, (m) presence of acute inflammatory process, (n) who had the previous experience with WBV prior to participating in the experiment.

**Figure 1:**
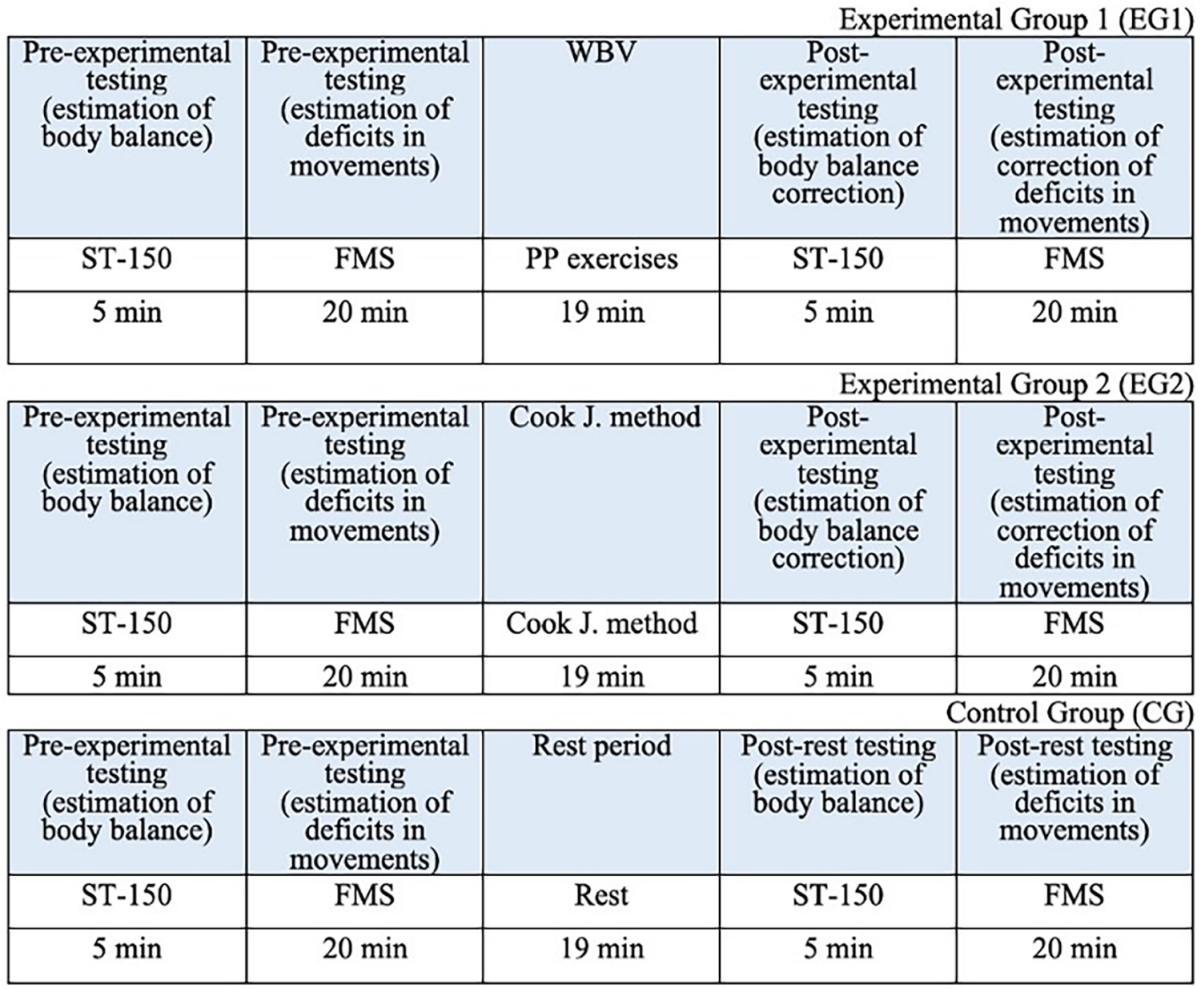
Study design.

The conditions for performing FMS tests and WBV exercises on the Power Plate platform were wearing the sportswear and shoes. To reduce the risk of sliding, non-slip rubber mat of 10 mm was attached to the platform surface. During the stabilomentry measurements, the participants were barefoot. The average time for performing the stabilometric trials, FMS tests, WBV exercises and FMSE are shown in the study design (Figure 1).

### 2.2 Study protocol (procedures)

#### 2.2.1 Pre-experimental stage

##### 2.2.1.1 Procedure of Functional Movement Screen

All subjects (*n* = 70) at the pre-experimental stage were tested by FMS, which involved performing seven fundamental functional movements and three clearing tests (47, 48). In total seven FMS test were analyzed: Deep Squat, Hurdle Step, In-Line Lunge, Shoulder Mobility, the Active Straight Leg Raise, the Trunk Stability Push-up and Rotary Stability. Each task was carried out using the standard FMS measurement kit (1.2 m bar, 2 cm by 60 cm bar, 5 cm by 15 cm box) (https://www.FunctionalMovement.com, Danville, VA, USA). FMS scoring was performed using the ordinal scale from “0” to “3”, which means that ordinal scale contained 4 values, “0 “- pain was reported during the movement, “1”—the inability to perform the movement, “2”—minor deficits or perfect performance with modifications; “3”—perfect performance. Five FMS tests examined both the right and left sides which were scored. The lower score of the two sides was recorded and counted toward the total. Three FMS tests have additional clearing screens (Shoulder Clearing test, Spinal extension clearing test, Spinal flexion clearing test) that are graded as positive or negative. Each test is performed to observe a pain response. If pain is produced, then positive is recorded on the score sheet and a score of zero is given for the associated test. The maximum total FMS score that can be attained is twenty-one. Two experienced evaluators gave grades, and the result was an average of three grades.

##### 2.2.1.2 Procedure of stabilometry

**Figure 2:**
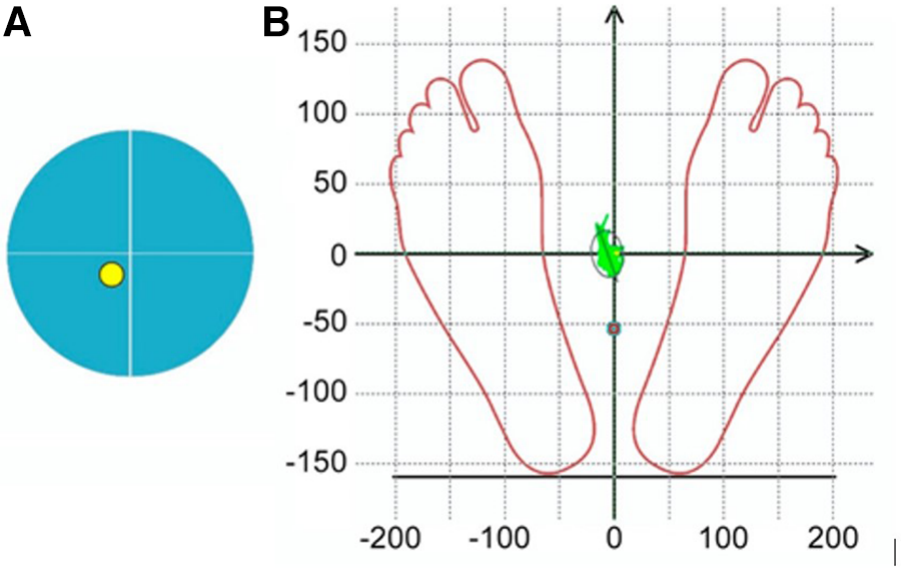
Marker symbols on the monitor screen for visual biofeedback during stabilometric tests: (**A**) during the Static test, it was recommended to keep the yellow COP marker within the blue circle on the monitor screen, (**B**) during the Given Setting test it was recommended to hold the COP color marker on the monitor screen as close as possible to the point of intersection of the *X*/*Y* axes.

To assess the body balance all subjects (*n* = 70) performed three pre-experimental stabilometric tests: Stance in Given Setting Test, Static Test (both in 30-s measurement interval) and two-phase Romberg Test (the eyes open and eyes closed, in 30-s measurement interval/each phase). Biofeedback was applied to stabilometric tests, by which the subjects controlled the COP position on the monitor screen using a color marker. Biofeedback sensitivity progressively increased during the Static Test, making it more difficult for the participant to hold the COP color marker inside the blue circle on the monitor screen. During the Given Setting Test, subjects were instructed to hold the COP color marker on the monitor screen as close as possible to the point of intersection of the *X*/*Y* axes (Figure 2). Stabilometry trial was performed on a Mera ST-150 stabilometric platform (Mera-TSP, Russia www.stpl.pro). The code of the STPL software in the international Global Medical Device Nomenclature system is: Posturography system application software 43115. During the measurements, the room was silent, and no other activities were performed in the room that could distract the measurement. The room temperature was comfortable for the participant's attire. The measured and assessment stabilometric parameters according to the research protocol are showed in the Table 2.

**Table 2:** Measured and assessment stabilometric parameters.

Parameter	Unit	Description
V	mm/s	Average rate of change in the position of the COP.
SD	%	The standard deviation of the pressure center^a^ relative to the ideal position.
Area	mm^2^	Area of the statokinesiogram^b^ with a confidence interval of 95%.
Length	Mm	Length of an ellipse containing 95% of the statokinesiogram’ points.
Width	Mm	Width of an ellipse containing 95% of the statokinesiogram’ points.
Max *X*	Mm	Maximum amplitude of the COP oscillations along the *X* axis.
Max *Y*	Mm	Maximum amplitude of the COP oscillations along the *Y* axis.
A	J	Work on moving the COP.
Av	mJ/s	Average rate of change in the work of moving the COP.
Am	mJ/kg	Work on moving the COP without considering the mass.

^a^ Center of Pressure (COP) is the point corresponding to the resultant of the body's pressure forces on the stabilometric platform. The coordinates of the pressure center at each moment of time are the primary data of stabilometry.

^b^ Statokinesiogram is the trajectory of the center of pressure, the totality of all points where the pressure center was located during registration.

#### 2.2.2 Experimental stage

##### 2.2.2.1 Procedure of Whole Body Vibration

**Figure 3:**
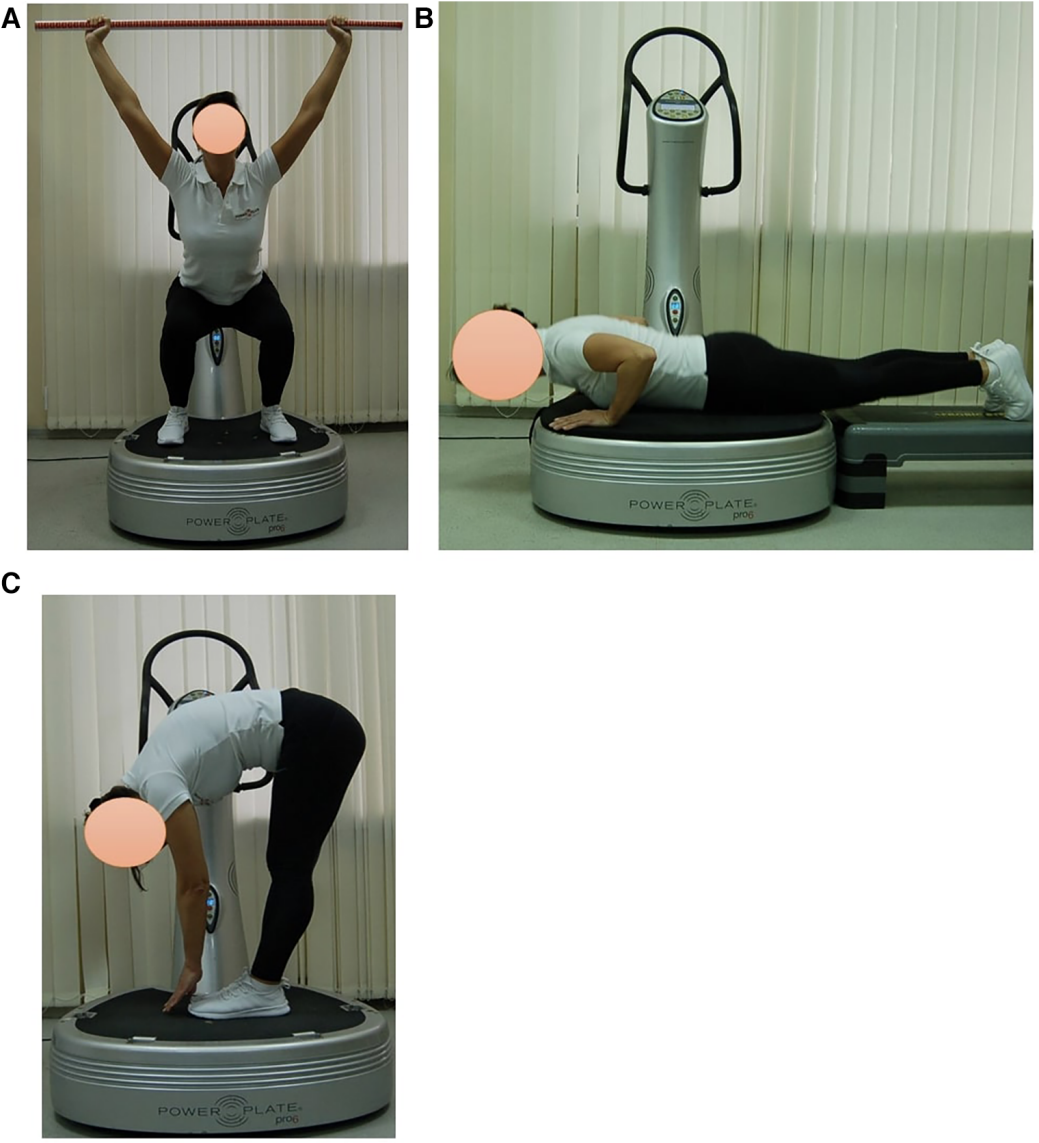
Examples of оne-side WBV exercises performed on the Power Plate platform. During one WBV session subjects of EG1 perform one exercise in the positions: (**A**) Deep Squat, (**B**) Trunk Stability Push-up, (**C**) Active Straight Leg. Muscle static reflex activities during WBV: Deep Squat—leg muscles; Trunk Stability Push-up—arms, shoulders and pectoralis muscles; Active Straight Leg—back leg stretching. The positions of the exercises correspond as closely as possible to the FMS tests in name and form of execution.

**Figure 4:**
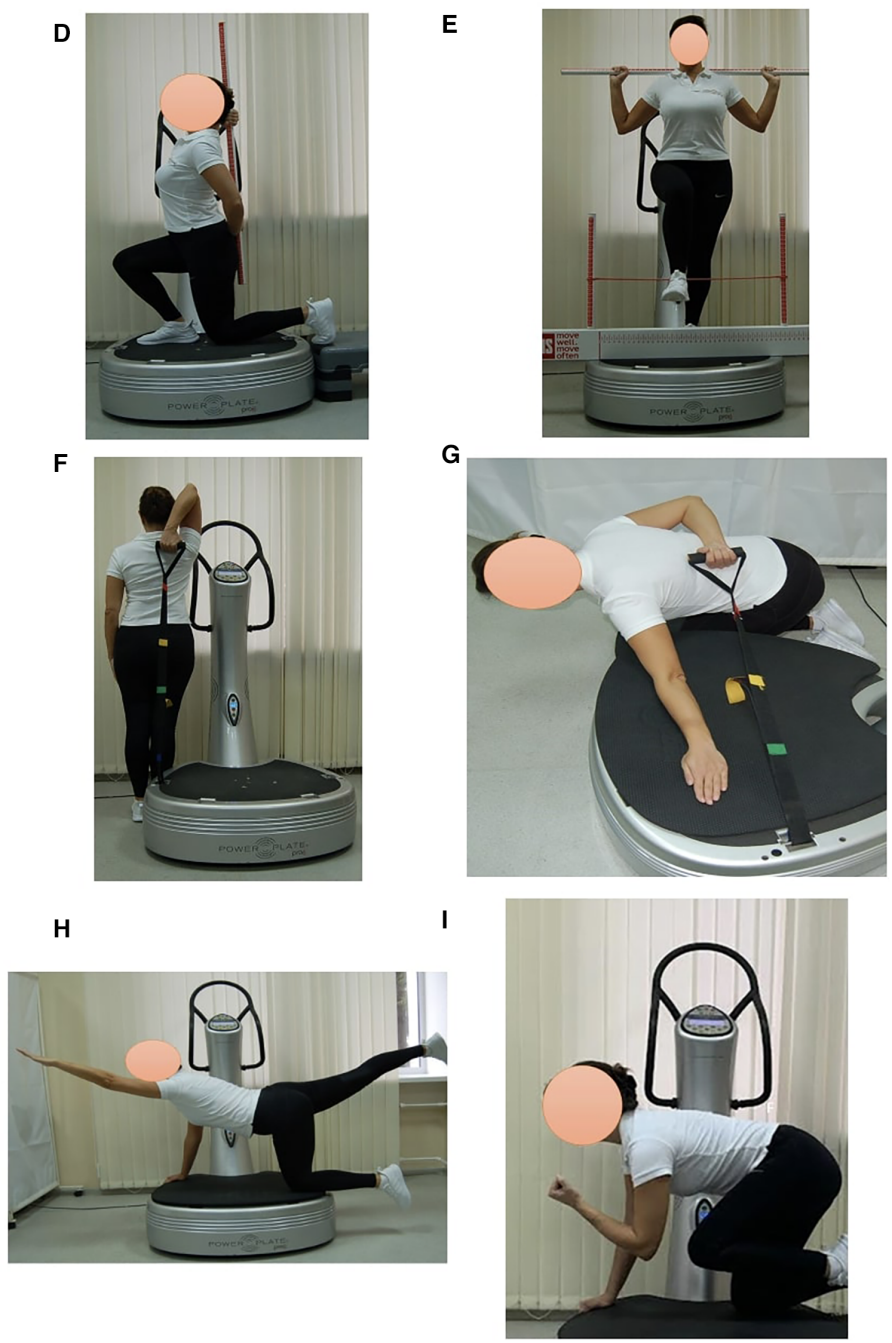
Examples of two-side WBV exercises performed on the Power Plate platform. During one WBV session subjects of EG1 perform one exercise in the positions: (**D**) In-Line Lung (right/left); (**E**) Hurdle Step (right/left); (**F**) Shoulder Mobility, exercise 1 (right/left); (**G**) Shoulder Mobility, exercise 2 (right/left); (**H**) Rotary Stability, exercise 1 (right/left); (**I**) Rotary Stability, exercise 2 (right/left). Muscle static reflex activities during WBV: In-Line Lung—leg and pelvic muscles; Hurdle Step—leg muscles; Shoulder Mobility—arm muscles, shoulder muscle stretch; Shoulder Mobility—arm muscles, shoulder muscle stretch; Rotary Stability—intermuscular coordination, leg, arm, gluteal and back muscles; Rotary Stability—intermuscular coordination, leg, arm, gluteal and back muscles. The positions of the exercises correspond as closely as possible to the FMS tests in name and form of execution.

Subjects in EG1 (*n* = 30) performed a single session of the WBV targeted static exercises adapted in the performance form to seven FMS tests on the vertical Power Plate Pro5 platform (Power Plate North America, Chicago, IL, USA). The Power Plate Pro5 was 87 cm × 109 cm × 157 cm height, has 158 kg mass, platform size was 87 cm × 94 cm. The vibration platform provided a constant vibration. Parameters of Power Plate workout was: frequency—35 Hz; amplitude platform movement—2 mm; platform acceleration—18 m/s^2^. Both the amplitude and frequency were set using the electronic platform settings. The platform was always switched on after subjects stood on it, and they stepped off after the device was turned off. The WBV sessions were carried out individually in the research laboratory of Neurosciences Research Institute of Samara State Medical University. All WBV sessions on the Power Plate platform were supervised by certified Power Plate Master Trainer. The WBV exercises adapted to FMS tests “Deep Squat”, “Trunk Stability Push-up” and “Active Straight Leg Raise” were one-side performed (Figure 3). The WBV exercises adapted to FMS tests “Hurdle Step”, “In-Line Lunge”, “Shoulder Mobility” and “Rotary Stability” were two-side performed (Figure 4). Thus, the number of the WBV exercises performing by every subject during one session was fifteen. Duration of a single WBV exercise was 45 s. The total session duration of WBV for every subject was 11 min. The rest time between the exercises was 30 s. The total rest period between the exercises during one WBV session for every subject was 8 min. Thus, the duration of one WBV session was 19 min.

##### 2.2.2.2 Procedure of Functional Movement Screen Exercises

Subjects in EG2 (*n* = 30) performed a single session of FMSE which was designed according to the standardized corrective algorithm suggested by Cook et al. (49). Duration of a single FMSE in EG2 was 45 s, identical to the duration of a single WBV exercise on the Power Plate platform in EG1. The number of the FMSE performing by every subject was fifteen. Duration of a single exercise was 45 s. The total session period of the FMSE for every subject was 11 min. The rest time between the exercises was 30 s. The total rest period between the exercises during one FMSE session for every subject was 8 min. Thus, the duration of one FMSE session was 19 min.

##### 2.2.2.3 Control group

Subjects in CG (*n* = 10) did not perform any physical activity after completing the pre-experimental testing by FMS and stabilometry. Duration of the rest period was 19 min.

#### 2.2.3 Post-experimental stage

At the post-experimental stage all subjects performed the post-experimental testing by FMS and stabilometry immediately after the completing the interventions (WBV session in EG1, FMSE session in EG2) and resting period in CG. To assess the body balance all subjects performed three post-experimental stabilomentic tests: Stance in Given Setting Test, Static Test and two-phase Romberg Test.

### 2.3 Statistical analysis

Statistical data processing was performed by Statistica.12 software. Normality of distribution was checked using the Shapiro–Wilk, Kolmogorov–Smirnov, and Lilliefors criteria. Most of the studied parameters were characterized by a distribution other than normal. The Wilcoxon Matched Pairs Test was used to compare the results before and after the exercises within the groups. Mann–Whitney *U*-Test was used to compare the groups with each other. The significance level for this study was set at *p* < 0.05.

## 3 Results

The pre-experimental testing by FMS showed that all subjects in the three groups had the motor deficits and asymmetries in the fundamental movement patterns (Table 3). According to the FMS scores, all young adults performed the highest in the Deep Squat and Shoulder Mobility tests and, in contrast, the lowest in the Trunk Stability Push-Up test. Also, all subjects demonstrated functional movement deficits in the Hurdle Step, In-Line Lung, Active Straight Leg Raise и Rotary Stability tests according to the FMS assessment. The obtained FMS results of EG1 and EG2 participants became the basis for studying the acute effects of WBV and FMSE on the movement performance, respectively. Effect of WBV and FMSE on postural control of the EG1 and EG2 subjects was analyzed using a comparative pre- and post-experimental testing.

**Table 3:** Pre-experimental FMS measurements.

FMS	Body side	Groups		
		EG 1	EG 2	CG
		M ± e	M ± e	M ± e
Deep squat		2.8 ± 0.1	3.0 ± 0.0	3.0 ± 0.0
Hurdle step	R	2.7 ± 0.1	2.5 ± 0.1	2.5 ± 0.2
	L	2.5 ± 0.1	2.5 ± 0.1	2.5 ± 0.2
	W	2.5 ± 0.1	2.4 ± 0.1	2.4 ± 0.2
In-line lung	R	2.8 ± 0.1	2.8 ± 0.1	2.7 ± 0.2
	L	2.7 ± 0.1	2.8 ± 0.1	2.8 ± 0.1
	W	2.6 ± 0.1	2.7 ± 0.1	2.7 ± 0.2
Shoulder mobility	R	3.0 ± 0.0	3.0 ± 0.0	2.9 ± 0.1
	L	2.9 ± 0.0	2.9 ± 0.0	2.8 ± 0.1
	W	2.9 ± 0.0	2.9 ± 0.0	2.8 ± 0.1
Active straight leg raise	R	2.7 ± 0.1	2.7 ± 0.1	2.7 ± 0.2
	L	2.6 ± 0.1	2.7 ± 0.1	2.7 ± 0.2
	W	2.5 ± 0.1	2.7 ± 0.1	2.7 ± 0.2
Trunk stability push-up		2.3 ± 0.2	1.9 ± 0.2	2.1 ± 0.3
Rotary stability	R	2.3 ± 0.1	2.2 ± 0.1	2.6 ± 0.2
	L	2.1 ± 0.1	2.3 ± 0.1	2.6 ± 0.2
	W	2.0 ± 0.1	2.2 ± 0.1	2.6 ± 0.2
Total Score		17.7 ± 0.4	17.7 ± 0.3	18.3 ± 0.6

R, right side of the body; L, left side of the body; W, worst score; M ± e, arithmetic mean ± standard error of the mean.

### 3.1 Acute effect of WBV and FMSE on FMS scores

**Figure 5:**
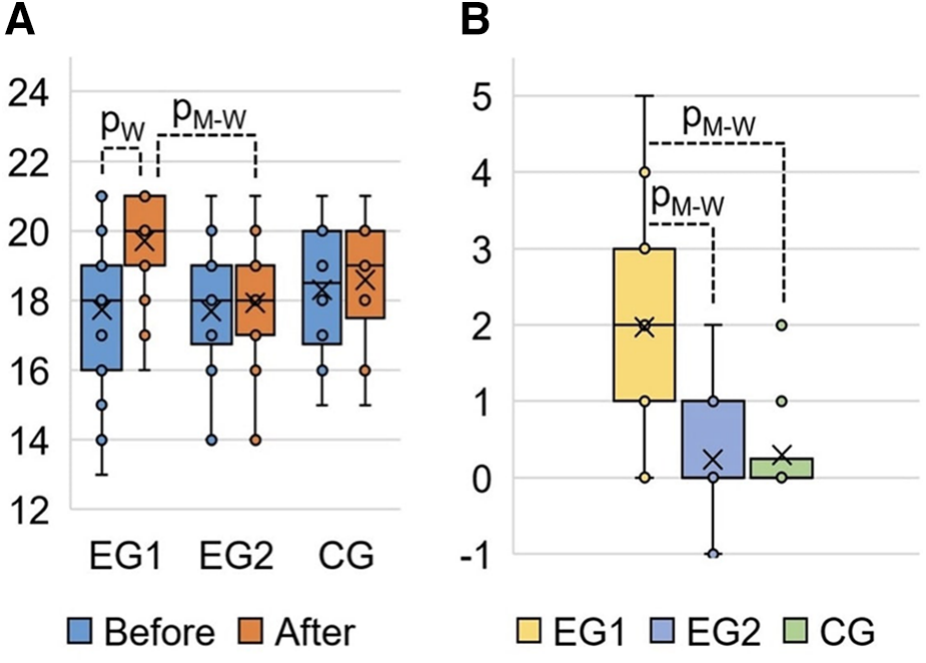
FMS total score in EG1, EG2 and CG: (**A**) FMS results at the pre-experimental stage (“Before”) and post- experimental stage (“After” the completing the WBV and FMSE interventions) measured in points; (**B**) change of the FMS total score at the post-experimental stage (“After”) compared to the pre-experimental stage (“Before”) measured in points. pW, calculated by Wilcoxon Matched Pairs Test (*р*_0_ < 0.001); pM-W, calculated by Mann–Whitney *U*-Test (*р*_0_ < 0.001).

The results of the post-experimental FMS testing showed a significant increasing in the sum of points (acute effect) after performing the WBV session, compared to the baseline data at the pre-experimental FMS testing in EG1 (Figure 5). Comparing the pre-experimental and post-experimental data in performance of each fundamental movement pattern in the FMS test in EG1 (Table 4), the positive dynamics was found concerning the following tests: Rotary Stability (0.6 ± 0.1; *p*_0_*_ _*< 0.001), Hurdle Step (0.4 ± 0.1; *p*_0_*_ _*= 0.002), In-Line Lunge (0.3 ± 0.1; *p*_0_*_ _*= 0.008), Active Straight Leg Raise (0.3 ± 0.1; *p*_0_*_ _*= 0.012) and Deep Squat (0.2 ± 0.1; *p*_0_*_ _*= 0.043). The total score in EG1 increased by 2.0 ± 0.2 (*p*_0_*_ _*< 0.001), аnd this was significantly differed (*p*_0_*_ _*< 0.001) from the EG2 and CG, where the changes were not statistically significant.

**Table 4:** Pre-experimental FMS measurements.

Statistical method			Post-experimetal change compared to pre-experimental value (*p*_0_ by Wilcoxon Matched Pairs Test)					Intergroup comparison (*p*_0_ by M–W *U* Test)		
Group		EG1		EG2		CG		EG1 vs. EG2	EG1 vs. СG	EG2 vs. СG
FMS	Body Side	M ± e	*p*_0_	M ± e	*p*_0_	M ± e	*p*_0_	*p*_0_	*p*_0_	*p*_0_
Deep squat		0.2 ± 0.1	**0**.**043**	0.0 ± 0.0	No Δ	0.0 ± 0.0	No Δ	0.271	0.444	0.988
Hurdle step	R	0.2 ± 0.1	**0**.**018**	0.0 ± 0.1	0.593	0.0 ± 0.1	1.000	0.204	0.333	0.901
	L	0.4 ± 0.1	**0**.**005**	0.1 ± 0.1	0.361	0.0 ± 0.0	No Δ	0.090	0.122	0.767
	W	0.4 ± 0.1	**0**.**002**	0.1 ± 0.1	0.225	0.1 ± 0.1	1.000	0.057	0.165	1.000
In-line lung	R	0.1 ± 0.1	0.109	0.0 ± 0.0	No Δ	0.1 ± 0.1	1.000	0.663	0.988	0.767
	L	0.2 ± 0.1	**0**.**012**	0.0 ± 0.0	No Δ	0.0 ± 0.0	No Δ	0.054	0.217	0.888
	W	0.3 ± 0.1	**0**.**008**	0.0 ± 0.0	1.000	0.1 ± 0.1	1.000	0.055	0.357	0.662
Shoulder mobility	R	0.0 ± 0.0	No Δ	0.0 ± 0.0	No Δ	0.0 ± 0.0	No Δ	0.830	0.888	0.988
	L	0.1 ± 0.0	0.180	0.0 ± 0.0	No Δ	0.0 ± 0.0	No Δ	0.830	0.767	0.888
	W	0.1 ± 0.0	0.180	0.0 ± 0.0	No Δ	0.0 ± 0.0	No Δ	0.830	0.767	0.888
Active straight leg raise	R	0.2 ± 0.1	**0**.**028**	0.1 ± 0.0	0.180	0.0 ± 0.0	No Δ	0.379	0.357	0.767
	L	0.3 ± 0.1	**0**.**018**	0.1 ± 0.0	0.180	0.0 ± 0.0	No Δ	0.264	0.281	0.767
	W	0.3 ± 0.1	**0**.**012**	0.1 ± 0.0	0.180	0.0 ± 0.0	No Δ	0.181	0.217	0.767
Trunk stability push-up		0.2 ± 0.1	0.068	0.0 ± 0.0	No Δ	0.0 ± 0.0	No Δ	0.511	0.542	0.888
Rotary stability	R	0.4 ± 0.1	**0**.**003**	0.0 ± 0.0	1.000	0.1 ± 0.1	1.000	**0**.**018**	0.212	0.662
	L	0.6 ± 0.1	**0**.**000**	0.0 ± 0.0	No Δ	0.1 ± 0.1	1.000	**0**.**000**	**0**.**030**	0.553
	W	0.6 ± 0.1	**0**.**000**	0.0 ± 0.0	1.000	0.1 ± 0.1	1.000	**0**.**000**	**0**.**020**	0.662
Total score		2.0 ± 0.2	**0**.**000**	0.2 ± 0.1	0.074	0.3 ± 0.2	0.180	**0**.**000**	**0**.**000**	0.988

R, right side of the body; L, left side of the body; W, worst score; M ± e, arithmetic mean ± standard error of the mean; *р*_0_, probability of null hypothesis; M–W *U* Test, Mann–Whitney *U*-Test.

The meaning of the bold values is “W” (for “Deep squat”, “Trunk stability push-up” and “Total score”).

### 3.2 Acute effects of WBV and FMSE on COP parameters

The acute effects of a single session of WBV and FMSE were analyzed by COP data in Static Test, Stance in Given Setting Test, and Romberg Test.

### 3.3 Rate of change and standard deviation (SD) of COP

In all groups when performing the Static Test after exercises the COP rate of change statistically significantly (*p*_0_*_ _*< 0.01) decreased (Table 5): in EG1 (after WBV) by 4.4 ± 2.4 mm/c; in EG2 (after FMSE) by 2.6 ± 0.6 mm/c; in CG (without experimental exposure) by 2.0 ± 0.6 mm/c.

**Table 5:** Rate of change in the position and standard deviation of the center of pressure.

Parameter	Statistical method		Сhange after exercises compared to the value before (*p*_0_ by Wilcoxon Matched Pairs Test)						Intergroup comparison (*p*_0_ by M–W *U* Test)		
	Group		EG1		EG2		CG		EG1 vs. EG2	EG1 vs. CG	EG2 vs. CG
	Test	Position	M ± e	*p*_0_	M ± e	*p*_0_	M ± e	*p*_0_	*p*_0_	*p*_0_	*p*_0_
V, мм/с	Static test		−4.4 ± 2.4	**0**.**004**	−2.6 ± 0.6	**0**.**000**	−2.0 ± 0.6	**0**.**007**	0.684	0.851	0.938
	Given setting test	Right control	0.5 ± 0.7	0.290	−0.6 ± 0.5	0.365	−0.7 ± 0.3	0.066	0.211	0.143	0.492
		Left control	0.4 ± 0.3	0.076	−0.3 ± 0.2	0.425	−0.1 ± 0.4	0.476	0.055	0.152	0.803
	Romberg test	Open eyes	0.6 ± 0.3	0.042	0.2 ± 0.2	0.184	0.1 ± 0.3	0.683	0.304	0.310	0.851
		Closed eyes	−0.5 ± 0.3	0.141	0.4 ± 0.4	0.491	−0.3 ± 0.4	0.575	0.098	0.708	0.399
SD, %	Static test		−2.3 ± 1.8	0.929	−2.0 ± 0.9	0.023	−6.1 ± 5.0	0.263	0.107	0.160	0.913
	Given setting test	Right control	4.7 ± 1.1	**0**.**000**	2.1 ± 0.6	**0**.**004**	−0.6 ± 0.6	0.281	**0**.**001**	**0**.**000**	**0**.**008**
		Left control	3.3 ± 0.6	**0**.**000**	0.8 ± 0.3	**0**.**009**	0.2 ± 0.4	0.673	**0**.**001**	**0**.**001**	0.049
	Romberg test	Open eyes	2.3 ± 0.6	**0**.**001**	1.4 ± 0.8	0.025	0.5 ± 0.5	0.407	0.067	0.080	0.492
		Closed eyes	1.1 ± 0.8	0.073	0.7 ± 0.5	0.082	0.5 ± 0.7	0.465	0.387	0.261	0.390

COP, center of pressure; V, average rate of change in the position of the COP; SD, the standard deviation of the COP relative to the ideal position; M ± e, arithmetic mean ± standard error of the mean; *р*_0_, probability of null hypothesis; M–W *U* Test, Mann–Whitney *U*-Test.

The meaning of the bold values “Position” is the word “Instruction” (for “Static Test”).

At the same time, there were no statistically significant differences in changes in this parameter between the groups (*p*_0_*_ _*> 0.65). In experimental groups, when performing the Given Setting Test after exercises, the SD parameter of the COP relative to the ideal position statistically significantly (*p*_0_*_ _*< 0.01) increased (Table 4): in EG1 (after WBV) in position «right control» by 4.7 ± 1.1% and in position «left control» by 3.3 ± 0.6%, in EG2 (after FMSE) in position «right control» by 2.1 ± 0.6% and in position «left control» by 0.8 ± 0.3%. The described SD changes statistically significantly (*p*_0_*_ _*< 0.01) distinguished EG1 from the other two groups in both positions, whereas EG2 statistically significantly differed from CG only in the “right control” position. Also, the SD parameter increased statistically significantly (*p*_0_*_ _*= 0.001) in EG1 when performing the Romberg Test after WBV in position “open eyes” by 2.3 ± 0.6%, whereas in other groups, when performing this test, the SD parameter did not show significant patterns.

### 3.4 Parameters of the confidence ellipse, containing 95% of the statokinesiogram's points

**Figure 6:**
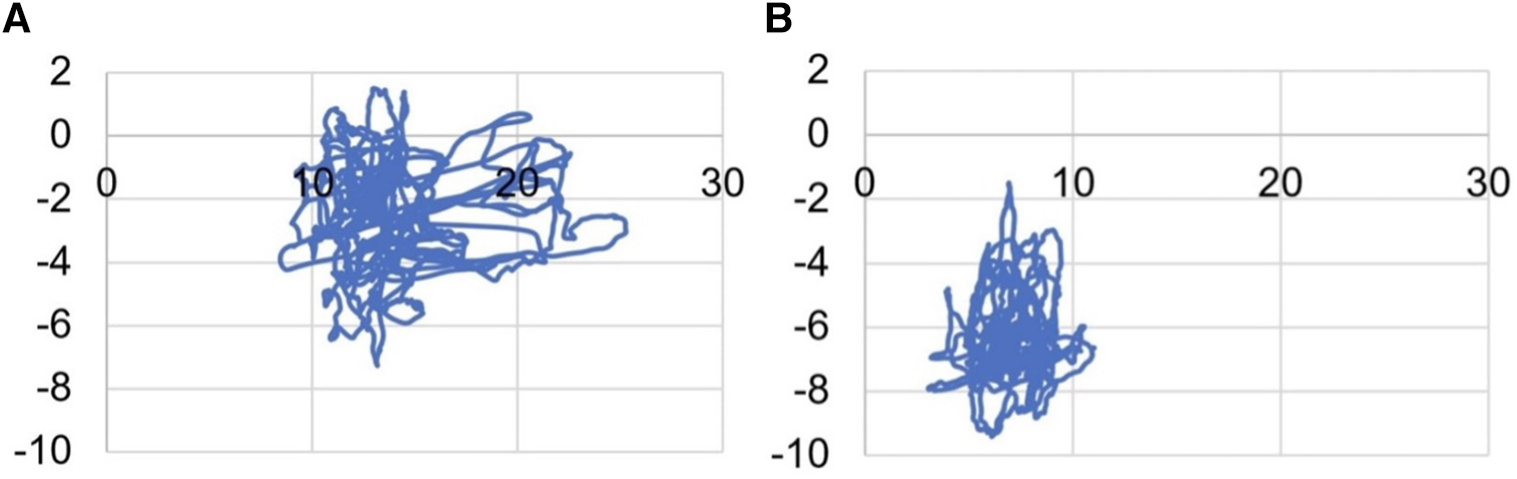
Change in statokinesiogram parameters in the Static test, mm: (**A**) before performing a single WBV session; (**B**) after performing a single WBV session.

In EG1 group, when performing the Static Test after WBV, the parameters of the ellipse, which includes 95% of the points of the statokinesiogram (Figure 6), statistically significantly (*p*_0_*_ _*< 0.01) decreased: length by 5.0 ± 2.2 mm, width by 6.2 ± 2.5 mm and area by 257 ± 170 mm^2^ (Table 5). In EG2 group, when performing the Static Test after FMSE statistically significantly (*p*_0_*_ _*< 0.01) decreased: width (by 4.2 ± 1.2 mm) and area (by 63.6 ± 21.7 mm^2^) of this ellipse. In EG1 group, when performing the Given Setting Test after WBV in position “right control” width of this ellipse statistically significantly (*p*_0_*_ _*< 0.01) decreased by 4.2 ± 1.4 mm. In CG group, when repeated execution the Romberg Test in position “open eyes” area of this ellipse statistically significantly (*p*_0_*_ _*< 0.01) increased by 5.2 ± 1.4 mm^2^.

### 3.5 Maximum amplitude of COP oscillations along the *X* and *Y* axes

The maximum amplitude of the COP oscillations (Table 6) when performing the Static Test after exercises in experimental groups statistically significantly (*p*_0_*_ _*< 0.01) decreased along the *X* and *Y* axes, and in CG—only along axis *Y*. In all groups when performing the Given Setting Test after exercises the maximum amplitude of the COP oscillations along the axis *Y* statistically significantly (*p*_0_*_ _*< 0.01) decreased: in EG1 in position “right control” by 3.2 ± 1.0 mm; in EG2 in position “left control” by 1.3 ± 0.4 mm; in CG in position “right control” by 2.8 ± 1.0 mm.

**Table 6:** Parameters of the confidence ellipse, containing 95% of the statokinesiogram's points.

Parameter	Statistical method		Сhange after exercises compared to the value before (*p*_0_ by Wilcoxon Matched Pairs Test)						Intergroup comparison (*p*_0_ by M–W *U* Test)		
	Group		EG1		EG2		CG		EG1 vs. EG2	EG1 vs. CG	EG2 vs. CG
	Test	Position	M ± e	*p*_0_	M ± e	*p*_0_	M ± e	*p*_0_	*p*_0_	*p*_0_	*p*_0_
Area, mm^2^	Static test		−257 ± 170	**0**.**002**	−63.6 ± 21.7	**0**.**001**	−44 ± 29	0.093	0.511	0.463	0.864
	Given setting test	Right control	−44.9 ± 38.3	0.061	−13.9 ± 12.1	0.318	−10.2 ± 11.4	0.646	0.222	0.368	0.988
		Left control	−4.7 ± 5.7	0.265	−9.7 ± 7.5	0.388	−11.6 ± 8.2	0.169	0.903	0.552	0.563
	Romberg test	Open eyes	16.5 ± 51.1	0.441	−4.2 ± 7.7	0.975	5.2 ± 1.4	**0**.**009**	0.520	0.864	0.864
		Closed eyes	−13.3 ± 18.6	0.558	13.5 ± 10.2	0.213	−1.2 ± 15.1	0.878	0.217	0.791	0.444
Length, mm	Static test		−5.0 ± 2.2	**0**.**002**	−2.0 ± 0.7	0.014	−1.0 ± 1.0	0.386	0.326	0.206	0.542
	Given setting test	Right control	−1.1 ± 1.2	0.596	−0.8 ± 0.7	0.191	−0.1 ± 0.7	0.878	0.928	0.860	0.482
		Left control	0.0 ± 0.5	0.959	−0.7 ± 0.5	0.447	−0.7 ± 0.8	0.286	0.688	0.629	0.803
	Romberg test	Open eyes	1.0 ± 1.1	0.600	−0.3 ± 0.5	0.434	0.8 ± 0.5	0.114	0.511	0.755	0.080
		Closed eyes	−1.0 ± 0.9	0.233	0.8 ± 0.5	0.341	−0.5 ± 0.9	0.508	0.139	0.815	0.241
Width, mm	Static test		−6.2 ± 2.5	**0**.**003**	−4.2 ± 1.2	**0**.**000**	−4.4 ± 2.3	0.022	0.941	0.963	0.963
	Given setting test	Right control	−4.2 ± 1.4	**0**.**007**	−0.4 ± 0.8	0.861	−1.5 ± 1.1	0.203	0.031	0.394	0.408
		Left control	−0.6 ± 0.6	0.304	−0.8 ± 0.5	0.206	−1.1 ± 0.7	0.173	1.000	0.563	0.502
	Romberg test	Open eyes	−0.5 ± 2.7	0.974	0.0 ± 0.9	0.537	0.1 ± 0.8	0.878	0.796	1.000	0.651
		Closed eyes	0.1 ± 1.2	0.918	0.5 ± 1.1	0.551	1.1 ± 1.4	0.333	0.652	0.502	0.673

M ± e, arithmetic mean ± standard error of the mean; *р*_0_, probability of null hypothesis; M–W *U* Test, Mann–Whitney *U*-Test.

The meaning of the bold values “Position” is the word “Instruction” (for “Static Test”).

### 3.6 Calculated energy consumption for moving of COP

Calculated parameters reflecting energy consumption for COP moving (Table 7) when performing the Static Test after exercises statistically significantly (*p*_0_*_ _*< 0.01) decreased in all groups: the work on COP moving decreased in EG1 (after WBV) by 31.2 ± 25.4 J, in EG2 (after FMSE) by 6.3 ± 2.2 J, in CG by 4.3 ± 1.9 J. The rate of change in the work of COP moving decreased in EG1 by 519 ± 423 mJ/s, in EG2 by 105 ± 37 mJ/s, in CG by 71 ± 31 mJ/s. The work on COP moving without considering the mass decreased in EG1 by 445 ± 363 mJ/kg, in EG2 by 90 ± 32 mJ/kg, in CG by 61 ± 27 mJ/kg.

**Table 7:** Maximum amplitude of the pressure center oscillations along the *X* and *Y* axes.

Parameter	Statistical method	Сhange after exercises compared to the value before (*p*_0_ by Wilcoxon Matched Pairs Test)							Intergroup comparison (*p*_0_ by M–W *U* Test)		
	Group		EG1		EG2		CG		EG1 vs. EG2	EG1 vs. CG	EG2 vs. CG
	Test	Position	M ± e	*p*_0_	M ± e	*p*_0_	M ± e	*p*_0_	*p*_0_	*p*_0_	*p*_0_
Max *X*, mm	Static test		−9.0 ± 3.1	**0**.**000**	−3.1 ± 0.9	**0**.**000**	−9.7 ± 7.0	0.173	0.107	0.310	0.755
	Given setting test	Right control	−1.1 ± 1.5	0.673	−0.3 ± 0.5	0.173	−0.3 ± 0.5	0.374	0.940	0.949	0.864
		Left control	0.1 ± 0.4	0.683	0.0 ± 0.4	0.975	−0.1 ± 0.5	0.575	0.756	0.664	0.767
	Romberg test	Open eyes	1.2 ± 0.7	0.168	0.5 ± 0.4	0.304	0.4 ± 0.5	0.508	0.663	0.755	0.988
		Closed eyes	−0.9 ± 0.6	0.139	0.3 ± 0.3	0.586	−0.3 ± 0.7	0.959	0.112	0.435	0.827
Max *Y*, mm	Static test		−7.0 ± 2.1	**0**.**001**	−7.2 ± 1.6	**0**.**000**	−6.9 ± 2.5	**0**.**009**	0.668	0.864	0.803
	Given setting test	Right control	−3.2 ± 1.0	**0**.**003**	−0.7 ± 0.6	0.313	−2.8 ± 1.0	**0**.**009**	0.044	0.974	0.044
		Left control	−0.5 ± 0.5	0.581	−1.3 ± 0.4	**0**.**008**	−1.0 ± 0.5	0.114	0.156	0.143	0.988
	Romberg test	Open eyes	−0.2 ± 0.8	0.561	0.5 ± 0.5	0.294	0.0 ± 0.7	0.959	0.240	0.606	0.606
		Closed eyes	−0.9 ± 0.9	0.727	0.8 ± 1.0	0.600	0.1 ± 0.9	0.646	0.647	0.988	0.963

Max *X*, maximum amplitude of the COP oscillations along the *X* axis; Max *Y*, maximum amplitude of the COP oscillations along the *Y* axis; COP, center of pressure; M ± e, mean ± standard error of the mean; *р*_0_, probability of null hypothesis; M–W *U* Test, Mann–Whitney *U* Test.

The meaning of the bold values “Position” is the word “Instruction” (for “Static Test”).

## 4 Discussion

The aim of this study is to investigate the functional role of proprioceptive system in motor patterns and body balance in conventionally healthy young adults. To achieve this goal, the study was performed using WBV technology according to the guidelines for WBV studies in humans (63).

It has previously been shown that the vibration effect on muscles is a strong proprioceptive stimulus, and the resulting proprioceptive activation induces muscle tuning and directly reaches the primary somatosensory and motor cortex (64). The concept of muscle tuning during oscillatory influences is fundamental in understanding the effects of sport enhancement (65, 66) and equally considered in the paradigm of WBV performance being dependent on changes in frequency and/or amplitude of the vibration machine (1, 16, 27, 67, 68).

Until now the paradigm of sport enhancement and WBV has been viewed from the perspective of the muscle tuning hypothesis (1, 65, 66). For the first time this article proposes a new paradigm—the integrative movement tuning hypothesis—which is very relevant for WBV and understanding the improvement of movement patterns, the control of which is based on the integrative function of the proprioceptive system. Our study demonstrated, according to the integrative movement tuning hypothesis, that the different levels of activation of the proprioceptive sensory system after the acute WBV session effectively improve functional motor patterns and COP in conventionally healthy adults.

According to the literature, there is a large evidence base of WBV research indicating the main targets of the WBV application, which include the CNS and musculoskeletal system (1, 11). Our study used the comprehensive methods to assess functional motor patterns and body balance using FMS (47, 48) and stabilometry. In terms of the stated aim of the study, the integration of FMS and stabilometry is due to the fact that the nervous control of the motor patterns and body balance have integrative levels of regulation and are closely interconnected (69–71). Sensorimotor control reflects the complex processes of integration into the CNS of information from the visual, vestibular and proprioceptive systems, the results of which influence motor patterns of movements (71) and balance (69).

As shown by the data of this study, the proprioceptive sensory system plays a key role in the interaction of the integrative levels of regulation of functional motor patterns and body balance (69–71). Moreover, numerous WBV studies show the integrative role of the M1 sensorimotor system (72). Early reports already showed that at the segmental level, the integration of spinal cord neural networks under the WBV influence is manifested by *γ*-motoneuron excitability, muscle coactivation, spindle sensitivity and synchronization (67).

That's why, in our study, WBV in the acute stimulation paradigm was an activator of the proprioceptive sensory system. This approach allows us to achieve maximum effect at the segmental control level of the activity of different threshold motor units and the motor pattern as a whole (16, 67). Unlike all other WBV studies, in our work the WBV static exercises in the executive form were as close as possible to the form of the FMS static exercises (47, 48). It should be emphasized that sensorimotor integration during the static WBV exercises is carried out under conditions of high-frequency stimulation of skeletal muscle proprioceptors and high-frequency reflex contractions (35 Hz × 45 s = 1.575 muscle contractions for each of the 15 WBV exercises). Moreover, it is known that with WBV muscle activation increased with the enhanced vibration frequency (19) in conditions of recruitment of high- and low-threshold motor units and different contributions of mono- and polysynaptic pathways of the spinal cord (16).

The segmental level of integration of proprioceptive afferentation during WBV includes the tonic vibration reflex (16, 17), mono- and polysynaptic tendon reflexes (6, 18). At the electromyography level, this is manifested by an increase in electrical activity of muscles (19, 73) due to the recruitment of motor units (16).

As a result of our study, FMS scores of pre- and post- acute WBV session in EG1 are significantly different (+2.0 ± 0.2; *p*_0_*_ _*< 0.001) (Table 3). In contrast, after a single FMSE session there was no change in FMS scores in EG2 (+0.2 ± 0.1; *р*_0_*_ _*< 0.074) (Table 4). Thus, the study established an acute positive WBV effect on FMS results in EG1 and the absence of such effect in EG2 after a short-term FMSE session (Table 4). Moreover, the acute WBV effects significantly influence the restoration of functional motor patterns in EG1 according to FMS results.

The results of COP in the Static Test and Given Setting Test (Table 5) after the acute WBV session of the static exercises adapted in the “mirror” form to FMS tests demonstrated the same positive trend.

It is important to emphasize that sensorimotor control of postural function is based on the complex integration of information from the visual, vestibular and proprioceptive systems, the interaction of which influences body balance (69). In addition, reactive postural control is a reflexive process that is significantly influenced by the requirements of a specific task, as well as the psychological state of the subject (74).

WBV is an important source of sensory flow to the sensorimotor cortex. The role of the cerebral cortex in postural control is to improve body balance, and many sensory inputs to the sensorimotor cortex are important, including inputs from skin exteroceptors (75) and the plantar cutaneous receptors (76, 77).

The cortical control of motor patterns and body balance involves motor commands from the M1 motor cortex, which has feedback between processes in M1 and multi-joint sensory integration (72). FMSE is controlled primarily by voluntary motor commands from the M1 motor cortex (72). In contrast, the acute WBV effects on postural control and motor patterns are realized through a more complex integration of different sensory streams. In our opinion, this is a significant difference between sensorimotor integration during WBV and FMSE and this can explain the high effectiveness of WBV on the FMS results in EG1 and the absence of dynamics of FMS score (Tables 3, 4) after a single FMSE session in EG2. Overall, the acute WBV effects on the recovery of motor patterns of FMS in EG1 and the absence of such effects after a single FMSE session (Table 4) can be explained by different levels of proprioceptive activation during WBV and FMSE.

On the other hand, the COP results according to the Static Test, Given Setting Test and Romberg Test after a single session of WBV and FMSE are noteworthy. Postural stability is a key factor of human body balance and equilibrium. Analyzing variations in the CОP is the one way to quantify postural stability (55–57).

Analysis of COP parameters after an acute session of WBV and FMSE did not reveal inter-group differences in most stabilogram indicators (Tables 5–8). Consequently, despite the significant difference in sensory recruitment during WBV and FMSE, postural control is a process sensitive to regulatory stimuli of different functional significance. This may be due to the fact that postural control is provided by the functions of extensive neural networks of the brain (78) and, especially, by the interaction between the vestibular system and proprioception. In postural control, these systems provide an integrated process of sensor activation in the body (79). This conclusion is supported by studies that have demonstrated the effects of multimodal interventions on postural control. The range of such effects included both vibratory and acoustic stimulations (80) and direct effects on cortical structures using the transcranial direct current stimulation (81). It is interesting, according to some authors, WBV stimulation disrupts the body balance in the short term, but cortical activity may contribute to the formation of new synergies and modulation of muscle activity (82).

**Table 8:** Calculated energy consumption for moving the center of pressure.

Parameter	Statistical method		Сhange after exercises compared to the value before (*p*_0_ by Wilcoxon Matched Pairs Test)						Intergroup comparison *(p*_0_ by M–W *U* Test)		
	Group		EG1		EG2		CG		EG1 vs. EG2	EG1 vs. CG	EG2 vs. CG
	Test	Position	M ± e	*p*_0_	M ± e	*p*_0_	M ± e	*p*_0_	*p*_0_	*p*_0_	*p*_0_
A, J	Static test		−31.2 ± 25.4	**0**.**000**	−6.3 ± 2.2	**0**.**000**	−4.3 ± 1.9	**0**.**005**	0.900	0.815	0.988
	Given setting test	Right control	0.7 ± 0.7	0.218	−0.3 ± 0.3	0.320	−0.5 ± 0.2	0.017	0.131	0.048	0.155
		Left control	0.2 ± 0.2	0.150	−0.1 ± 0.1	0.299	−0.1 ± 0.2	0.333	0.088	0.267	0.651
	Romberg test	Open eyes	0.4 ± 0.3	0.106	0.1 ± 0.1	**0**.**020**	0.1 ± 0.1	0.575	0.501	0.512	0.719
		Closed eyes	−0.4 ± 0.3	0.107	0.3 ± 0.3	0.572	−0.2 ± 0.3	0.445	0.119	0.719	0.408
Av, mJ/s	Static test		−519 ± 423	**0**.**000**	−105 ± 37	**0**.**000**	−71 ± 31	**0**.**005**	0.912	0.815	0.988
	Given setting test	Right control	24.4 ± 23.2	0.222	−11.1 ± 8.5	0.329	−17.9 ± 5.6	**0**.**017**	0.128	0.052	0.146
		Left control	6.8 ± 5.8	0.144	−4.2 ± 3.5	0.329	−2.9 ± 7.9	0.333	0.083	0.267	0.673
	Romberg test	Open eyes	13.1 ± 9.5	0.094	4.0 ± 2.3	**0**.**020**	3.3 ± 4.1	0.575	0.464	0.502	0.719
		Closed eyes	−11.9 ± 10.3	0.125	9.3 ± 8.7	0.558	−5.6 ± 8.4	0.445	0.115	0.696	0.408
Am, mJ/kg	Static test		−445 ± 363	**0**.**000**	−90 ± 32	**0**.**000**	−61 ± 27	**0**.**005**	0.912	0.815	0.988
	Given setting test	Right control	10.5 ± 10.0	0.230	−4.8 ± 3.6	0.318	−7.7 ± 2.4	**0**.**017**	0.131	**0**.**048**	0.142
		Left control	2.9 ± 2.5	0.150	−1.8 ± 1.5	0.309	−1.2 ± 3.4	0.333	0.088	0.267	0.662
	Romberg test	Open eyes	5.6 ± 4.1	0.096	1.7 ± 1.0	**0**.**020**	1.4 ± 1.8	0.575	0.487	0.492	0.696
		Closed eyes	−5.1 ± 4.4	0.120	4.0 ± 3.7	0.565	−2.4 ± 3.6	0.445	0.122	0.731	0.408

COP, center of pressure; A, work on moving the COP; Av, arithmetic mean of the rate of change in the work of moving the COP; Am, work on moving the COP without considering the mass; M ± e, mean ± standard error of the mean; *р*_0_, probability of null hypothesis; M–W *U* Test, Mann–Whitney *U*-Test.

The meaning of the bold values “Position” is the word “Instruction” (for “Static Test”).

Our study shows that in the presence of functional deficits in postural control as measured by COP, acute exposure of WBV and FMSE can restore body balance in young adults. In addition, acute WBV represents a rapid method for teaching motor patterns and postural control skills (83).

Thus, in our study, FMS motor patterns and COP parameters in conventionally healthy young adults were the objects of a comprehensive study at the different levels of activation of the proprioceptive system of the brain, either using a short-term session of WBV static exercises or a short-term session of FMSE static exercise. Moreover, the WBV motor patterns were a “mirror” copy of the FMS motor patterns (49), which was done for the first time in the study. Overall, our study reveals the key role of the ascending proprioceptive system of the brain in the genesis of the acute effectiveness of WBV in restoring motor patterns and expands previously known ideas about the influence of WBV on the functions of the nervous system and muscular component of the musculoskeletal system (1, 11). We believe that the experimental paradigm of “mirror” integration of WBV with other methods of studying motor patterns for the purposes of carrying out fundamental and applied research, for an example, in sport, is promising. Literature analysis and data of this study give grounds to assert that the integrative movement tuning hypothesis regarding the integrative proprioceptive system of the brain explains the physiological meaning of the effectiveness of WBV technology.

## 5 Limitations

Further studies of electrical activity of skeletal muscle during different WBV and FMS motor patterns are needed to assess the extent of proprioceptive system involvement. It seems important to determine biomarkers of activation of the brain proprioceptive system, compositions of the subjects, levels of physical and psychophysiological status on the day of study. In terms of the work performed, further studies of forebrain areas in the paradigm of integrative movement tuning hypothesis are required.

## 6 Conclusions

The results demonstrate the statistically significant improvements in FMS tests and body balance performance after a single session of WBV static exercises, which are adapted in the performance form to the FMS tests. A comparative analysis of the FMS results carried out immediately after a single session of WBV and a single session of FMSE showed the statistically significant effectiveness of WBV in eliminating the motor deficits in young adults who were tested by the FMS method. In addition to the acute improvement of FMS scores by WBV, the study showed an acute positive effect of WBV on the stabilometric parameters. According to the integrative movement tuning hypothesis we formulate, the key property of the proprioceptive system, which is maximally manifested in WBV, is motor integrative learning of the brain's neural networks, which is demonstrated by FMS motor patterns. The absence of inter-group differences in indicators of body balance improvement after an acute session of WBV and FMSE indicates the high sensitivity of postural control and body balance to the different levels of integrative influence of the proprioceptive system. Our complex approach on base of the integrative movement tuning hypothesis can be considered as a new methodology of elimination the limitations in fundamental movement patterns and postural control.

## Data Availability

The raw data supporting the conclusions of this article will be made available by the authors, without undue reservation.
